# CL-ACP: a parallel combination of CNN and LSTM anticancer peptide recognition model

**DOI:** 10.1186/s12859-021-04433-9

**Published:** 2021-10-20

**Authors:** Huiqing Wang, Jian Zhao, Hong Zhao, Haolin Li, Juan Wang

**Affiliations:** College of Information and Computer, Taiyuan University of Technology, Taiyuan, 030024 China

**Keywords:** Anticancer peptide, Secondary structure, Neural network model, Attention mechanism

## Abstract

**Background:**

Anticancer peptides are defence substances with innate immune functions that can selectively act on cancer cells without harming normal cells and many studies have been conducted to identify anticancer peptides. In this paper, we introduce the anticancer peptide secondary structures as additional features and propose an effective computational model, CL-ACP, that uses a combined network and attention mechanism to predict anticancer peptides.

**Results:**

The CL-ACP model uses secondary structures and original sequences of anticancer peptides to construct the feature space. The long short-term memory and convolutional neural network are used to extract the contextual dependence and local correlations of the feature space. Furthermore, a multi-head self-attention mechanism is used to strengthen the anticancer peptide sequences. Finally, three categories of feature information are classified by cascading. CL-ACP was validated using two types of datasets, anticancer peptide datasets and antimicrobial peptide datasets, on which it achieved good results compared to previous methods. CL-ACP achieved the highest AUC values of 0.935 and 0.972 on the anticancer peptide and antimicrobial peptide datasets, respectively.

**Conclusions:**

CL-ACP can effectively recognize antimicrobial peptides, especially anticancer peptides, and the parallel combined neural network structure of CL-ACP does not require complex feature design and high time cost. It is suitable for application as a useful tool in antimicrobial peptide design.

**Supplementary Information:**

The online version contains supplementary material available at 10.1186/s12859-021-04433-9.

## Background

Antimicrobial peptides (AMPs) are natural and vital defence substances involved in innate immunity to certain diseases. Anticancer peptides (ACPs) are a class of antimicrobial peptides composed of 10–50 amino acids that have a killing effect on cancer cells [1]. ACPs interact with the cancer cell membrane and effectively destroy its structure, thereby inhibiting the proliferation and growth of cancer cells and inducing apoptosis [2, 3]. Studies have shown that ACPs have clear inhibition and elimination effects on cervical cancer cells, rectal cancer cells, and hepatocellular carcinoma cells [4]. ACPs can effectively remove cancer cells from the body and improve the body's immune function to resist invasion by tumour cells. Presently, many ACPs targeting different types of cancer have undergone clinical application [5–8]. Therefore, for the treatment and research of cancer, it is important to determine whether AMPs have antitumour activity, which would provide a new development direction for novel ACPs.

As small molecular peptides, ACPs have specific rules governing their peptide chains, and many researchers have conducted related studies [9, 10]. Hajisharifi et al. introduced peptide sequences as characteristic information to develop a model that successfully predicted ACPs [11]. To compensate for the uniqueness of peptide sequences, Tyagi et al. added information such as the dipeptide compositions and amino acid compositional differences between the N-terminal and C-terminal as features, achieving a higher accuracy than that of Hajisharifi [12]. Chen et al. combined the pseudo amino acid composition, mean chemical shift, and simplified amino acid composition to significantly improve ACP prediction accuracy [13]. However, these methods considered only original sequences and physicochemical properties of amino acids and neglected peptide structural characteristics at the spatial level.

Studies have shown that although the types and sequence length of ACPs vary, most ACPs contain characteristic structural information, such as α-helix or β-chain structures [14–16], which allow ACPs to act selectively on cancer cells [17]. With the development of protein structural property prediction techniques [18], researchers have begun to examine the role of protein structural information in AMPs. Chen et al. changed the hydrophobicity and amphiphilicity of peptide molecules by amino acid substitution in different regions of the peptide sequences [19, 20], which proved that the secondary structures play a critical role in the antitumour activity of ACPs. Based on the mechanisms of ACPs, Hammami et al. analysed the direct involvement of structural information in the formation of amphiphile side chains of ACPs [21]. They concluded that protein structural characteristics are the basis of the selective action of ACPs on cancer cells. Therefore, protein structural properties contain highly potent local and global features that provide strong evidence for the prediction of ACPs.

Most ACP prediction models are designed based on traditional machine learning algorithms such as Support Vector Machine (SVM), Random Forest (RF), Naive Bayesian (NB) or statistical models. Chen et al. used SVM to predict ACPs and achieved a high prediction accuracy [13]. Wei et al. used amino acid compositions and other information, combined with SVM to construct 40 submodels to predict ACPs and achieved good results [22]. In addition, some ACP prediction methods are based on the combination of multiple classifiers and the fusion of multiple sequence features [23–25]. Although these methods have made some achievements, the feature construction and extraction process is still tedious and depends on feature design and prior knowledge to some extent. In addition, the algorithm designs of these models are relatively complex, and their performances depend mainly on the number of feature types and the scale of the models.

A neural network can automatically learn advanced representation from raw data, providing a suitable means to solve the problems mentioned above. These networks have been successfully applied in many fields, such as image recognition, machine reading and bioinformatics [26–30]. Yi et al. predicted ACPs by integrating binary profile features and a k-mer sparse matrix with simplified amino acid identification and realised automatic feature extraction by long short-term memory (LSTM) to address the time-dependence problem in sequences [31]. Yi's work was the first attempt to apply a deep recurrent neural network (RNN) to predict ACPs. Wu et al. mapped peptide sequences to word vectors using the word2vec [32] tool and obtained multiangle features from different sizes of receptor fields using a text-convolutional neural network (text-CNN) [33, 34].

Feature extraction methods vary among different neural networks. LSTM automatically learns dependencies in sequence data through its memory units and gate mechanism. However, the limitation of its learning mechanism causes difficulty in learning local features in sequence data. As compensation, CNN compensate for this limitation by capturing local relevant features in input through convolution kernels. Therefore, a combined network can effectively improve model prediction abilities [35]. Wang et al. proposed a hybrid deep learning model for miRNA prediction based on integrating CNN and bidirectional long short-term memory (BILSTM) [27], which improved the prediction quality by capturing complex local features of nucleotides via CNN and long-term interdependence between nucleotides by BILSTM. Guo et al. developed DeepACLSTM by combining an asymmetric CNN and a BILSTM network to predict protein secondary structures effectively [28]. Therefore, the combination of CNN and LSTM can simultaneously focus on the local spatial and long-term dependence information in the original data, effectively reducing information loss and improving the ACP prediction performance.

**Fig. 1:**
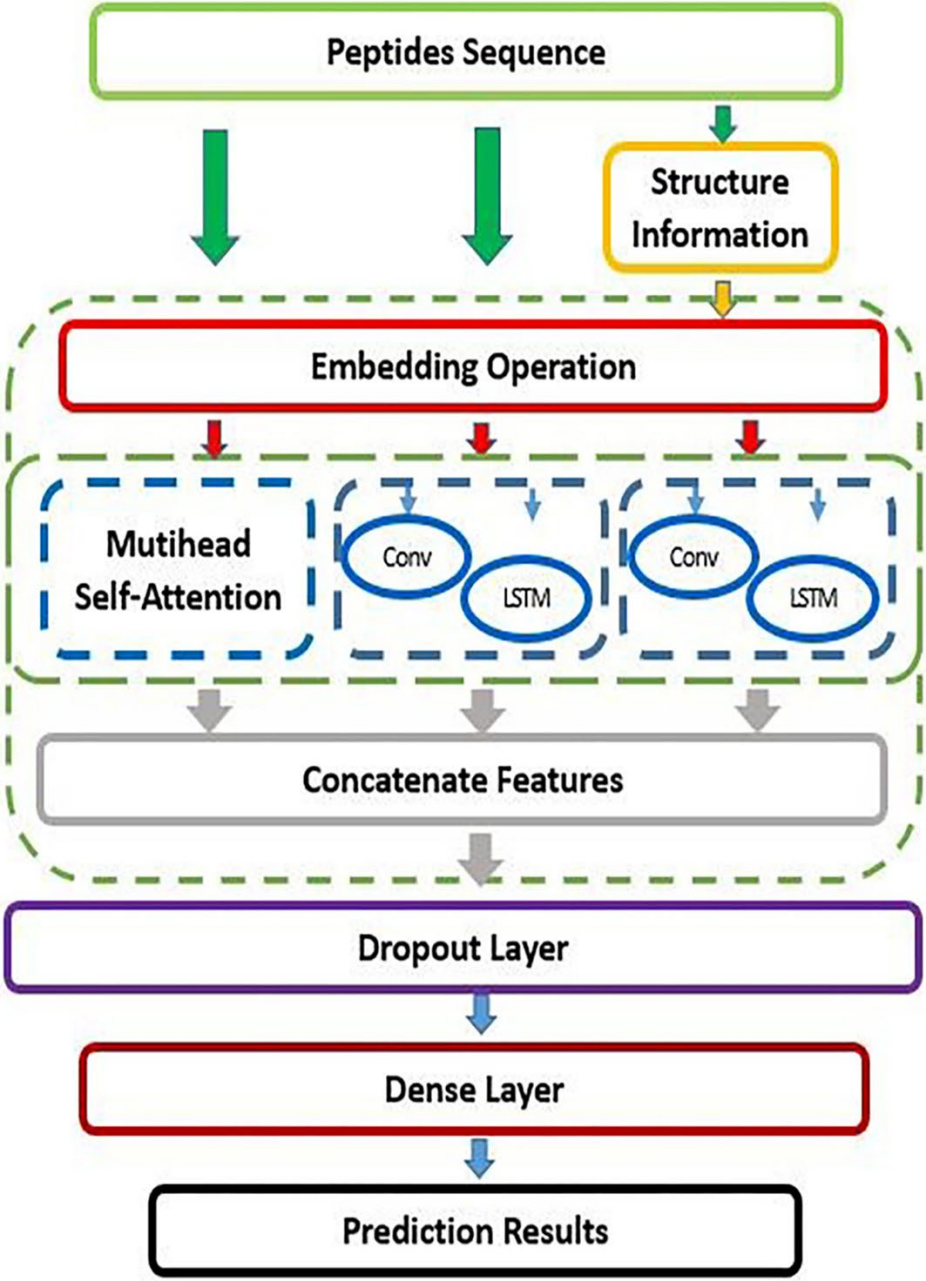
CL-ACP model framework

Based on the above problems, we effectively combined CNN and LSTM to propose a new neural network model, CL-ACP, for ACP recognition (Fig. 1). The CL-ACP model constructed a feature space from two aspects—ACP sequences and secondary structures. In addition, it used multi-head self-attention [36] to enhance peptide sequence representations. Finally, the CNN and LSTM parallel combined network model was applied to effectively capture the temporal and spatial feature information of peptide sequences and structural characteristics. To evaluate the predictive performance of CL-ACP, we conducted an experiment on the datasets of Yi et al. and compared CL-ACP with existing methods. The fivefold cross-validation experimental results show that CL-ACP can automatically learn the effective characteristics of complex correlation patterns in the data and further identify ACPs effectively.

## Methods

In this paper, we introduced the secondary structure information of ACPs and the multi-head self-attention mechanism and proposed a parallel combination of CNN and LSTM to predict ACPs. We evaluated the model predictive performance on the benchmark datasets ACP736 and ACP240 and compared it with machine learning and neural network models.

### Datasets

In this paper, we selected the ACP736 and ACP240 datasets collected by Yi et al. as the benchmark datasets and constructed a new dataset, ACP539. These ACP datasets contain the most experimentally verified positive samples in the literature thus far, as shown in Table 1.

**Table 1:** Summary of the scale of AMP datasets supplied in this work

Peptides	Number	Positive	Negative
AAP	214	107	107
ABP	1600	800	800
ACP240	240	129	111
ACP736	736	375	361
ACP539	539	189	350
CPP	740	370	370
QSP	400	200	200
PBP	160	80	80

Positive samples from the ACP736 and ACP240 datasets were experimentally verified, and negative samples were AMPs without anticancer functions. The benchmark datasets were all passed through the CD-HIT [37] tool to remove redundant sequences, with the threshold set to 0.9 to ensure that there were no duplicates. We also constructed a supplementary dataset, ACP539, to further verify the performance of the model. We generated positive samples by utilizing the previous works [12, 13, 22, 38] and the Antimicrobial peptide date (APD), which contain a total of 793 experimentally verified ACPs. For the collection of negative samples, we utilized AMPs and random peptides from the previous works [12, 38], wherein AMPs had been extracted from several databases including, the antimicrobial peptide date (APD), collection of anti-microbial peptides (CAMP) and database of anuran defense peptides (DADP), for which no anticancer activity has been reported in the literature. For random peptides, we assume that they are non-ACPs. Subsequently, we eliminated the duplicate samples of ACP739 and ACP240 from all the samples. To avoid performance over-estimation introduced by the homology bias, the remaining peptide sequences in both the positive and negative datasets with more than 90% sequence identity were removed using the CD-HIT program with the threshold set at 0.9. Finally, we obtained ACP539 dataset that contained 189 positive samples and 350 negative samples. The data volume ratio of positive and negative samples in ACP539 is approximately 1:2.

To verify the model generalizability, we selected other AMP datasets [39–45] to verify the prediction ability of the model for short peptide data. The AMP datasets are also shown in Table 1.

### Extraction of peptide secondary structures

ACPs can target cancer cells because of their special mechanism of membrane cleavage. The main mechanism representations are the bucket plate model, blanket model, and annular pore model [46–48]. In the disintegration of the cancer cell membrane, the bucket plate and annular pore models form ionic channels penetrating the plasma membrane, which require at least three peptide molecules with a particular secondary structure. Relevant studies have proven that many ACPs exert their anticancer effects through the bucket plate and annular hole models. For example, melittin, which was discovered in 1994, has anti-inflammatory, analgesic, antibacterial, anti-HIV, antitumour, and other pharmacological activities. It also has a broad spectrum of antitumour activities, including against human hepatocellular carcinoma, leukaemia, and breast cancer [49, 50]. ACPs from *Litoria raniformis* have strong effects on leukaemia, lung cancer, colon cancer, and other diseases [51]. *Magainins* from *Xenopus laevis* can destroy the cell membrane of human cervical cancer cells through annular pore action [52]. Therefore, the action mechanism of most ACPs is based on their secondary structures, and the accurate extraction of peptide secondary structures thus plays a vital role.

To accurately extract peptide secondary structures, we analysed the performances of various secondary structure predictors and selected the SPIDER3 [18]. We also eliminated four peptide sequences that lacked secondary structure from the ACP736 dataset.

### Representation of the features

The input feature space of CL-ACP was composed of three kinds of feature information, including peptide original sequences and secondary structures encoded by one-hot coding [53] and peptide sequences enhanced by the multi-head self-attention mechanism.

#### One-hot coding and multi-head self-attention mechanism

ACPs are usually composed of 5–40 amino acids of 20 different types. Each amino acid was encoded using one-hot coding of length 20, representing 20 dimensions corresponding to the 20 amino acids. Thus, an original sequence of length L was encoded as a vector representation of dimension L*20. The structure information included three exponents: α-helix P (H), β-chain P (C), and γ-strand P (E). Therefore, we obtained a vector representation of L*3-dimensional protein structure property information for motifs of length L.

Although one-hot coding is a simple and effective coding method, its premise is to assume that coding elements are independent of each other; however, residues in peptide sequences are not independent of each other. Moreover, the proportions of certain residues in peptide sequences are relatively high, which indicates that these residues play relatively important roles in antitumour activity. Obviously, using one-hot coding alone cannot reflect the degree of distance between elements and fully characterize sequences. To strengthen the expression of peptide sequences and extract high-quality feature information, we introduced a multi-head self-attention mechanism to focus on the relatively important residues in the sequences. The multi-head self-attention mechanism is a variant of the attention mechanism, which has been widely used in tasks such as machine reading, text summarization, and image description. Compared with the self-attention mechanism, multiple heads can form multiple subspaces, allowing the attention mechanism to evaluate the importance of residues from different subspaces [54]. To the best of our knowledge, this paper introduces the multi-head self-attention mechanism into peptide sequences coding for the first time. The input sequence vectors are calculated by the multi-head self-attention mechanism to obtain new characterization vectors, allowing the model input to represent more context information. Moreover, the multi-head self-attention mechanism associates any two amino acid residues in the sequences by calculating the similarity between the elements without limiting the distance between them. Therefore, it does not need to fix the length of peptide sequences and can dynamically adjust the weights of different amino acids in sequences to preserve complete feature information.

In addition, to select a suitable number of heads in the multi-head self-attention mechanism, we set the numbers of heads as 1, 2, 4, 8 and 16 and evaluated the proposed model performance, as shown in Additional file 2: Table S1. Compared with the model with a multi-head self-attention mechanism, the comprehensive performance of the model using the common self-attention mechanism (only 1 head) was relatively poor, which indicates that the multi-head self-attention mechanism can comprehensively evaluate the importance of residues in sequences from multiple perspectives. Moreover, the number of heads is an important hyperparameter in the multi-head self-attention mechanism, and the number of heads is not necessarily proportional to the effect of the model [55]. The results in Table S1 show that when the number of heads increased from 2 to 16, the performance of the model decreased. After we added regularization to each head [56], this phenomenon improved to some extent. As shown in Additional file 3: Table S2, when the number of heads was large, more redundant subspaces were generated, leading to high similarity between heads. Although heads regularization can increase the diversity among multiple attention heads, but it also increased time cost when the number of heads was large. Therefore, we selected 2 as the optimal number of heads to avoid serious similarity problems among heads, and the resulting model had the best comprehensive performance and a low time cost.

The multi-head self-attention mechanism contains multiple identical self-attention structures, and each attention head uses different initialization parameters to learn different attention spaces. The self-attention mechanism uses scaled dot-product attention to calculate similarity scores. The calculation of similarity scores is shown in Eq. 1.

1 $$Score = \frac{{Query*Key^{T} }}{{\sqrt {d_{w} } }}$$

where $Query$ represents an amino acid and $Key$ represents each amino acid in a peptide sequence, $d_{w}$ represents the word vector dimension, and $Score$ represents the similarity between the evaluated amino acid and each amino acid in a peptide sequence.

The similarity scores are then normalized by softmax and converted into a probability distribution with the sum of weights equals to 1, thus highlighting the correlation between the two elements, as shown in Eq. 2.

2 $$\alpha = Softmax\left( {Score} \right)$$

Finally, the attention score of the current amino acid is obtained by multiplying the normalized similarity score by the current amino acid. The calculation process is shown in Eq. 3.

3 $$Attention\left( {Query,Key,Value} \right) = \alpha \cdot Value$$

In the multi-head self-attention mechanism, $Value$ represents the same value as $Query$*.*

$Query$, $Key$, and $Value$ are mapped to multiple parallel heads for repeated attention calculations through different parameter matrices. Each head can process different information, and the calculation process is shown in Eq. 4.

4 $$head_{i} = Attention\left( {Query \cdot W_{i}^{Query} ,Key \cdot W_{i}^{Key} ,Value \cdot W_{i}^{Value} } \right)$$

The weight parameters $W_{i}^{Query} \in R^{{d_{w} /h*d_{w} }}$, $W_{i}^{Key} \in R^{{d_{w} /h*d_{w} }}$ and $W_{i}^{Value} \in R^{{d_{w} /h*d_{w} }}$ are learnable parameters for linear calculation.

The multi-head self-attention mechanism can process different parts of a sequence to extract richer sequence features and combine the results of multiple attention operations into vector stitching, as shown in Eq. 5.

5 $$MultiHead\left( {Query,Key,Value} \right) = W^{M} \left[ {head_{1} ,head_{2} , \cdots head_{h} } \right]$$

where *h* is the number of parallel heads, and $W^{M} \in R^{{d_{w} *d_{w} }}$ is used to connect several attention results, which can maintain the original output dimension. The final calculation process of the multi-head self-attention mechanism is shown in Eq. 6.

6 $$S^{\prime } = \left\{ {w_{1}^{\prime } ,w_{2}^{\prime } , \ldots ,w_{n}^{\prime } } \right\} = MultiHead\left( {S,S,S} \right)$$

In the multi-head self-attention mechanism, $Query$, $Key$, and $Value$ all represent the original sequence S, and ${\text{S}}^{\prime }$ is the output. ${\text{w}}_{{\text{i}}}$ is a new output of amino acids in the sequence calculated by the multi-head self-attention mechanism, which contains richer sequence information. The final ${\text{S}}^{\prime } \in {\text{R}}^{{{\text{n*d}}_{{\text{w}}} }}$ is a new representation of a peptide sequence.

### The framework of CL-ACP

In this paper, we propose CL-ACP to effectively predict potential ACPs. To prevent cross-talk between the peptide original sequences and secondary structures, we used two sets of parallel CNN and LSTM composite structures to extract the features respectively, and then combined the extracted features with the enhanced sequence features of multi-head self-attention to obtain advanced features. Finally, the advanced features were input to a fully connected layer to predict ACPs.

#### Convolutional neural network

Due to the different characteristics of the sequence information carried by the original sequences and secondary structures, we introduced two sets of single-layer two-dimensional convolutional neural networks to extract features from the two types of information, with each branch consisting of a convolutional layer and rectifying linear unit (ReLU). The convolutional layer can obtain local features by convolving the sequences encoding space and rectifying linear elements to sparse the convolution layer output. Due to the short length of the peptide sequences, we did not pool the features after convolution, thus preserving the feature integrity.

The convolutional layer of peptide sequence and structure information consists of 300 and 150 convolution kernels, and the sizes of the convolution kernels are 5*5 and 3*3, respectively. The convolution kernels are convolved with the input peptide sequences to output a series of weight numbers indicating the convolution kernel matching degree with each window. The inner product of the output matrix of the convolutional layer is shown in Eq. 7.

7 $$C_{\imath } = \mathop \sum \limits_{b = 1}^{8} \mathop \sum \limits_{j = 1}^{20} K_{b,j} X_{b,j + 1}$$

where $X \in \left\{ {0,1} \right\}^{T*L}$ is the input matrix after encoding, *T* is the number of different elements in the sequences, *K* is the convolution kernel of 5*5 or 3*3, and $C _{l}$ eliminates the negative matches in the convolutional layer and maintains positive matches by ReLU processing. Finally, the original sequences and the convolution branch of the secondary structures are stitched and input to the fully connected layer.

#### Long short-term memory network

To identify category information hidden in the original and secondary structure sequences, we added LSTM, which incorporates long-term dependence information to aid in prediction. As the LSTM scans each element of the input sequences, first, the forget gate determines what information to discard based on the previous input. Then, the input gate determines how much new information should be added to the cell state to update the current state value. Finally, the output gate arranges the values to determine which values to output. These gating operations enable the LSTM to automatically extract and learn all relevant information from the sequences that is useful for the overall classification task.

The numbers of storage units in the LSTM hidden layer of CL-ACP are 45 and 20 for feature extraction in the original sequences and secondary structures, respectively. The gating mechanism of LSTM and the update state of each step are shown in Eqs. 8–12.

8 $$i_{t} = \sigma \left( {W_{xi} x_{t} + W_{hi} h_{t - 1} + W_{ci} c_{t - 1} + b_{i} } \right)$$

9 $$f_{t} = \sigma \left( {W_{xf} x_{t} + W_{hf} h_{t - 1} + W_{cf} c_{t - 1} + b_{f} } \right)$$

10 $$c_{t} = f_{t} c_{t - 1} + i_{t} \tanh \left( {W_{xc} x_{t} + W_{hc} h_{t - 1} + b_{c} } \right)$$

11 $$o_{t} = \sigma \left( {W_{xo} x_{t} + W_{ho} h_{t - 1} + W_{co} c_{t} + b_{o} } \right)$$

12 $$h_{t} = o_{t} \tanh \left( {c_{t} } \right)$$

where $\sigma$ is the sigmoid function, and *i*_*t*_, *f*_*t*_, *o*_*t*_ and *c*_*t*_ represent the input gate, forget gate, output gate and cell activation vector, respectively. *X, h*, and *c* represent input vectors, hidden states, and memory locations, respectively. *W* and *b* are weights and offsets that need to be learned. We selected the sigmoid function as the activation function, and Eq. 13 shows the calculation process.

13 $$\sigma = sigmoid\left( x \right) = \frac{1}{{\left( {1 + e^{ - x} } \right)}}$$

Simultaneously, we chose the corresponding binary cross-entropy loss function in the binary classification tasks to adjust the neural network. Equation 14 is the definition of the loss function.

14 $$logloss\left( {t,p} \right) = - \left( {\left( {1 - p} \right) \times \log \left( {1 - p} \right) + t \times \log \left( p \right)} \right)$$

where *P* and *T* represent the predicted and target values of the model, respectively. Finally, the *Adam* optimizer commonly adopted in the neural network was used to update the network weight.

Considering the limited ACP data and thcomplex network model structure that may lead to overfitting problems, we used dropout [57] and early stopping regularization methods to optimize the model and reduce the model parameters. The loss rate p was set to 0.45, and dropout was only used during training. In addition, the CNN, LSTM and the multi-head self-attention mechanism were combined in parallel to reduce the number of network layers, model complexity and time consumption and maintain the richness of feature dimensions.

#### Performance evaluation criteria

We considered several statistical measures to aluate the performance of the proposed model and other comparative models, including accuracy (Acc sensitivity (Sens), specificity (Spec), precision (Prec) and Matthew’s correlation coefficient (Mcc). Thr definitions are shown in Eqs. 15–19.

15 $$Acc = \frac{TP + TN}{{TP + FN + TN + FP}}$$

16 $$Sens = \frac{TP}{{TP + FN}}$$

17 $$Spec = \frac{TN}{{TN + FP}}$$

18 $$Prec = \frac{TP}{{TP + FP}}$$

19 $$Mcc = \frac{TP \times TN - FP \times FN}{{\sqrt {\left( {TP + FP} \right) \times \left( {TP + FN} \right) \times \left( {TN + FN} \right) \times \left( {TN + FP} \right)} }}$$

where *TP* denotes true positives, *TN* denotes true negatives, *FP* denotes false positives, and *FN* denotes false negatives. Acc measures the total number of correctly identified ACPs and non-ACPs. Sens evaluates the accuracy of the model in identifying ACPs. Spec assesses the ability of predictors to recognize non-ACPs. Prec evaluates the number of correctly predicted ACPs in the identified data. When positive and negative samples are unbalanced, Mcc can measure the classification quality of a classifier. In addition, the area under the receiver operating characteristic (ROC) curve (AUC) was used to measure the overall performance of the model. The higher the values of these indicators are, the better the overall performance of the model.

## Results

### The performances of CL-ACP on the benchmark datasets

To evaluate the CL-ACP model ACP predictive ability, we conducted fivefold cross-validation on the benchmark datasets ACP736 and ACP240. Detailed information about the fivefold cross-validation experiment on the benchmark datasets is shown in Table 2.

**Table 2:** The 5-fold cross-validation details in the ACP datasets

Fold	Acc(%)	Sens(%)	Spec(%)	Prec(%)	Mcc(%)
ACP736					
1	83.78	84.00	83.56	84.00	67.56
2	83.00	85.33	80.55	82.05	66.00
3	85.03	82.67	87.50	87.32	70.19
4	84.35	86.67	81.94	83.33	68.73
5	82.99	76.00	90.28	89.06	66.82
Average	83.83	82.93	84.76	85.15	67.86
ACP240					
1	89.58	96.15	81.82	86.21	79.45
2	81.25	92.31	68.18	77.42	63.02
3	89.58	84.62	95.45	95.65	79.86
4	91.67	84.62	99.89	99.89	84.62
5	87.50	96.00	78.26	82.76	75.86
Average	87.92	90.74	84.76	88.41	76.56
ACP539					
1	87.04	81.58	90.00	81.58	71.58
2	80.56	65.79	88.57	75.76	56.36
3	83.33	74.37	88.41	78.38	63.52
4	89.81	84.62	92.75	86.84	77.82
5	81.31	81.08	81.43	69.77	60.64
Average	84.41	77.48	88.23	78.46	65.98

**Fig. 2:**
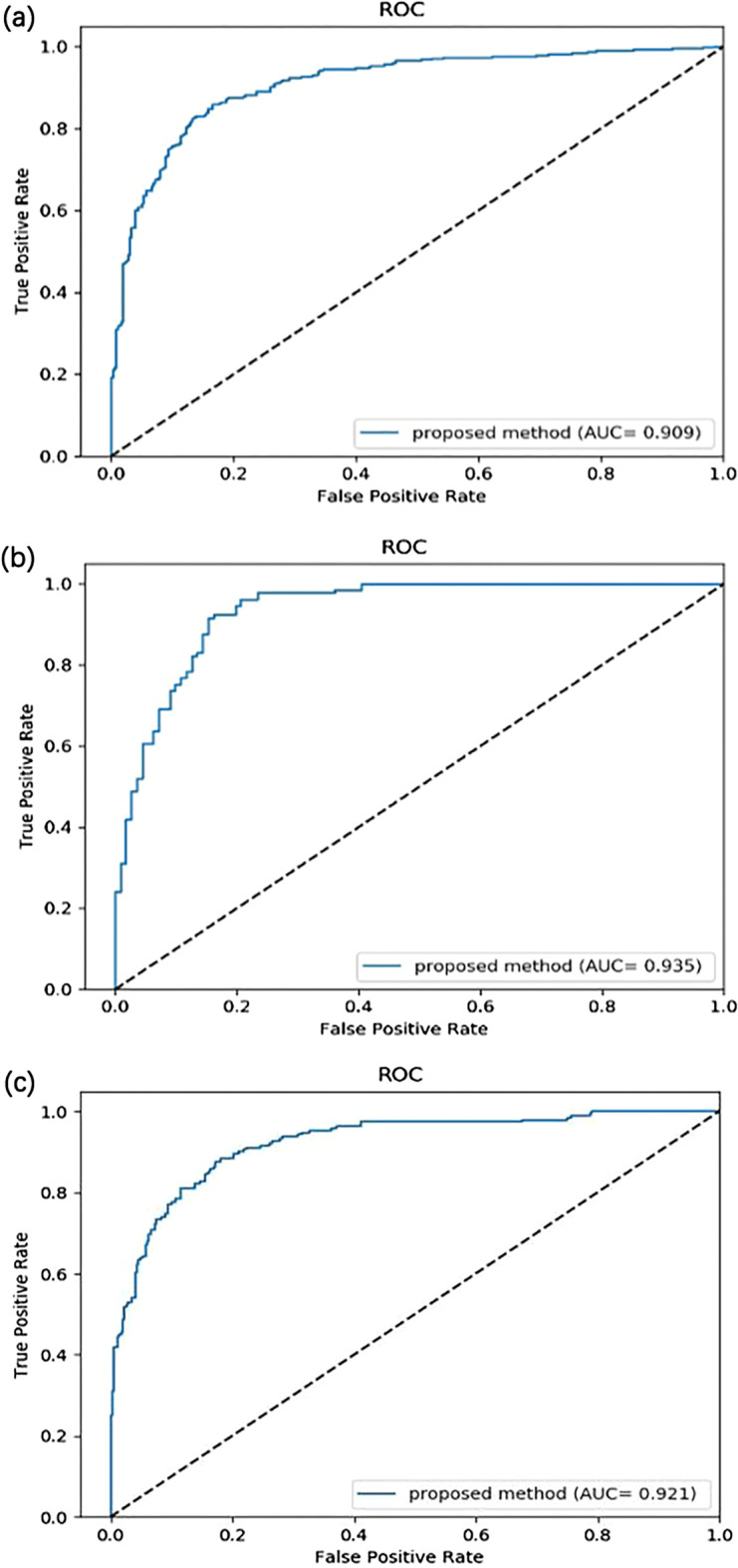
ROC curves of CL-ACP on the ACP datasets. **a** ROC curve of the CL-ACP model on ACP736. **b** ROC curve of the CL-ACP model on ACP240. **c** ROC curve of the CL-ACP model on ACP539

The average Acc of fivefold cross-validation on ACP736 was 83.83%, the average Sens was 82.93%, the average Spec was 84.76%, the mean Prec was 85.15% and the average Mcc was 67.86%. CL-ACP had an AUC of 0.909, as shown in Fig. 2a. On dataset ACP240, the average Acc of fivefold cross-validation was 87.92%, the average Sens was 90.74%, the mean Spec was 84.72%, the average Prec was 88.41%, the average Mcc was 76.56%, the AUC was 0.935, and the ROC curve is shown in Fig. 2b. The CL-ACP model achieved a good prediction performance on both datasets, especially on the smaller ACP240 dataset, indicating that CL-ACP has good ACP prediction ability and robustness.

To further verify the CL-ACP model performance, we conducted experiments on the newly constructed ACP539 dataset. The ratio of positive samples to negative samples in the ACP539 dataset is 1:2. Table 2 shows the fivefold cross-validation results of CL-ACP on ACP539. As Table 2 shows, on dataset ACP539, the average Acc of fivefold cross-validation was 84.41%, the average Prec was 78.46%, the average Sens was 77.48%, the average Spec was 88.23%, and the average Mcc was 65.98%. The AUC was 0.921, and the ROC curve is shown in Fig. 2c. The results of fivefold cross-validation show that the accuracy, specificity, and ROC value of CL-ACP on the ACP539 dataset were promising. However, the accuracy and sensitivity were lower than those on the two benchmark datasets, mainly because the ACP539 dataset was slightly unbalanced and contained noise in the negative samples.

In addition, to analyse the robustness of the proposed model, we further performed k-fold cross-validation, setting k = 6, 8 and 10. The results are shown in Additional file 4: Table S3. From Additional file 4: Table S3, we can see that there was no significant fluctuation among the index values with different values of k. The congruence of k-fold cross-validation results indicates the promising performance and robustness of CL-ACP.

### Ablation experiments

To verify the vital role of protein structural characteristic information in predicting ACPs and each CL-ACP component's necessity, we used fivefold cross-validation to conduct ablation experiments on the benchmark datasets. The procedure mainly included the introduction of two-dimensional CNN, the use of multi-head self-attention mechanism, and the introduction of peptide secondary structures. The experimental results are shown in Table 3. The baseline model used the original sequence information of the motifs as input and applied LSTM to extract features and predict ACPs.

**Table 3:** Ablation experiment results on the benchmark datasets

Component	LSTM	√	√	√	√	√
	CNN		√	√	√	√
	Multi-SA (skip-connection)			√		√
	Structure information				√	√
	Multi-SA(cascade)				√	
ACP736	Acc (%)	78.94	82.88	83.55	83.55	**83.83**
	Sens (%)	78.66	80.06	82.26	83.20	**82.93**
	Spec (%)	79.21	80.62	82.82	83.94	**84.76**
	Prec (%)	80.04	82.12	84.00	84.54	**85.15**
	Mcc (%)	58.12	65.84	67.37	67.27	**67.86**
	AUC	0.862	0.897	0.904	0.900	**0.909**
ACP240	Acc (%)	83.33	85.66	86.25	85.83	**87.92**
	Sens (%)	88.43	89.93	90.00	86.89	**90.74**
	Spec (%)	77.55	81.00	82.05	84.74	**84.76**
	Prec (%)	82.77	84.01	86.34	87.86	**88.41**
	Mcc (%)	67.83	73.03	73.18	72.23	**76.56**
	AUC	0.867	0.915	0.920	0.914	**0.935**

Bold indicates the results of the final model (CL-ACP)

**Fig. 3:**
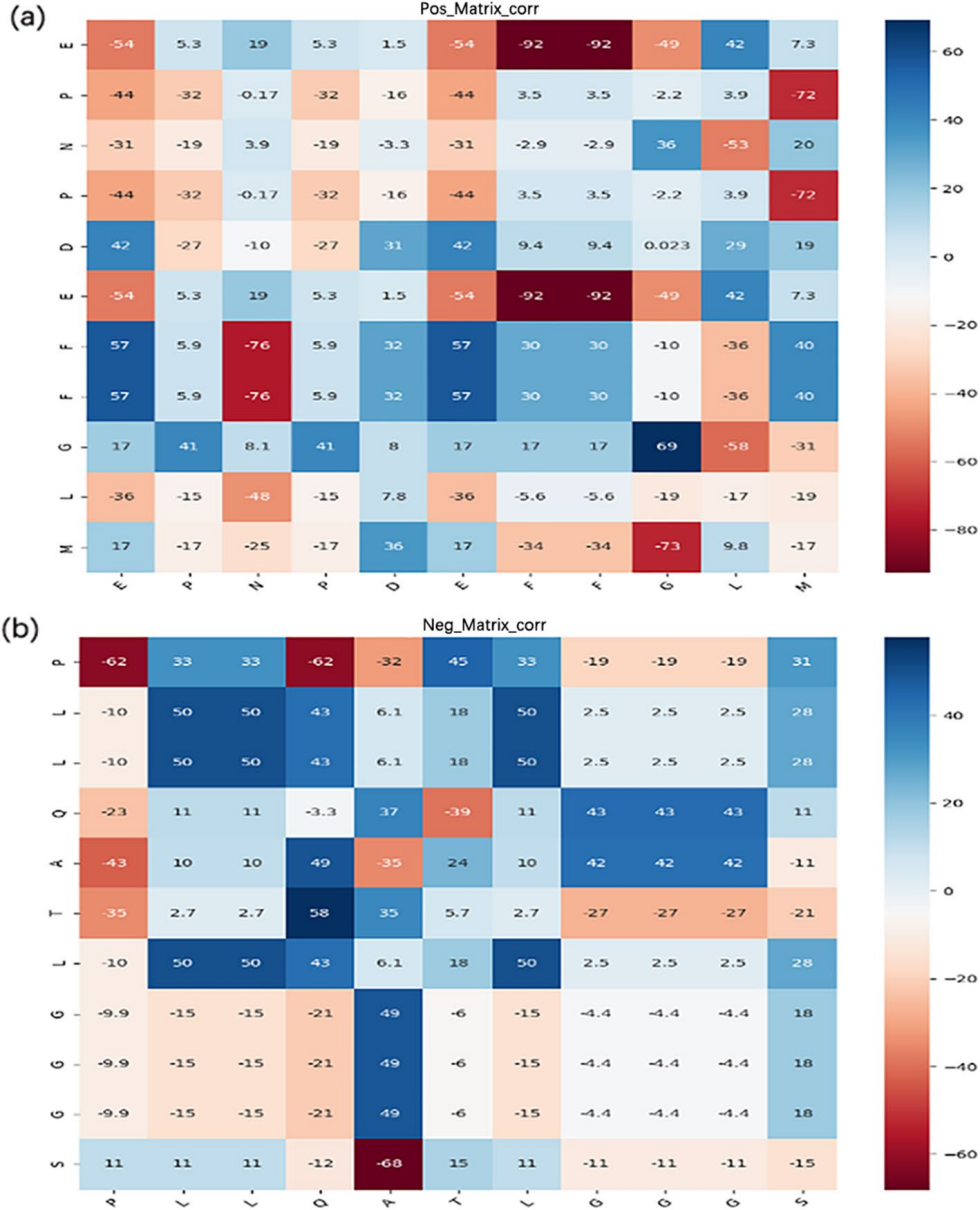
Self-attention weights of ACP and non- ACP. **a** Multi-head self-attention weight diagram of ACP. **b** Multi-head self-attention weight diagram of non-ACP

The introduction of two-dimensional convolution dramatically improved all indicators of the baseline model for ACP736 and ACP240, as shown in the fourth column of Table 3. The improvements show that the two-dimensional convolutional network can capture spatial feature information in peptide sequences. Compared with the model using LSTM alone, the CNN and LSTM parallel combined structure can fully extract sequence feature information from multiple angles and obtain a higher-quality abstract representation. In the fifth column of Table 3, we found that the introduction of a multi-head self-attention mechanism improved multiple indicators of the model. These improvements show that the multi-head self-attention mechanism can focus the model on more critical residue information in peptide sequences and strengthen the peptide character representations, thereby enhancing the representation ability of the network. Yi’s work indicated that the contents of amino acids Cys (C), Phe (F), Gly (G), His (H), Ile (I), Asn (N), Ser (S), and Tyr (Y) accounted for a significantly higher proportion of ACPs than non-ACPs. However, the amino acids Glu (E), Leu (L), Met (M), Gln (Q), Arg (R), and Trp (W) accounted for a greater proportion in non-ACPs than ACPs. Visualization shows that the multi-head self-attention mechanism could effectively capture essential features of peptide sequences, as shown in Fig. 3. In the multi-head self-attention visualization diagram of randomly selected an ACP, the weights of amino acids Phe and Gly with respect to the whole sequence (the blue row in the matrix) are larger than those of other amino acids, as shown in Fig. 3a. Similarly, in randomly selected a non-ACP, the weight of the amino acid Leu in the whole sequence was higher than those of other amino acids, as shown in Fig. 3b. These visualizations are consistent with the findings of the previous study [31]. Considering that ACPs are relatively short, the model combination will not only yield more abundant characteristic information but also increase the input noise and useless information. Therefore, we added the multi-head self-attention mechanism to the fully connected layer by skip connection. The skip connection reduced the parameters and time cost of the model and improved the model efficiency. As shown in the sixth and seventh columns of Table 3, the performance of cascading multiple self-attention mechanism into a parallel network was poorer than that of using a skip connection. In addition, as shown in Additional file 5: Table S4, the number of parameters in the cascading mode was 2.16 times that of the skip connection mode, and the running time of the former was approximately 4 times that of the skip connection mode. These results show that the introduction of skip connection is effective. As shown in the seventh column of Table 3, the addition of peptide secondary structures caused a significant increase in the model indicators. Compared with using original sequence information alone, the values of Acc, Sens, Spec, Prec, Mcc and ROC on ACP736 increased by 0.15%, 0.77%, 0.94%, 1.39%, 0.21% and 0.5%, respectively. Similarly, the values of Acc, Sens, Spec, Prec, Mcc and ROC on ACP240 increased by 1.67%, 0.74%, 2.71%, 2.07%, 3.38%, and 1.5%, respectively. Compared with the model without secondary structure information, all model indicators significantly improved after secondary structure information was added, with most improved by approximately 2%. These improvements indicate that ACP secondary structures contain critical information about antitumour activity. The introduction of ACP secondary structures enriches the feature space and facilitates ACP identification.

## Comparison with other classification models on benchmark datasets

To further assess the prediction performance of the proposed model, we compared it on the benchmark datasets with other models, including SVM, RF, NB, AntiCP2.0, ACP-DL, PTPD and iACP-DRLF models [24, 25]. The first four models are machine learning methods, and the last three models are neural network methods. Among them, AntiCP 2.0 and iACP-DRLF only provide web servers or trained models; thus, we used the data from each fold in the fivefold cross-validation to evaluate the trained model and took the average value as the result. This verification method was also used in related works [58, 59]. In addition, iACP_DRLF provided two trained models, with one trained by a dataset composed of ACPs and non-ACPs and the other trained by a dataset composed of ACPs and AMPs, which were denoted as iACP_DRLF(a) and iACP_DRLF(b), respectively. Similar to iACP_DRLF, AntiCP2.0 also provided two trained models, AntiCP2.0(a) and AntiCP2.0(b). We validated all four models on our ACP datasets, and the detailed results are shown in Additional file 6: Table S5. Considering the similar compositions of the datasets, we chose iACP_DRLF(a) and AntiCP2.0(a) for comparison with the proposed method using the baseline datasets and iACP_DRLF(b) and AntiCP2.0(b) for comparison with the proposed method using ACP539. All methods were evaluated with the same evaluation indicators. The comparison results are shown in Table 4. On the dataset ACP736 dataset, the specificity of CL-ACP was lower than that of the NB model, but the other indicators of CL-ACP were the highest. The NB sensitivity and other indicators were lower than those of the other models. On dataset ACP240, all the indicators of CL-ACP were the highest. Overall, CL-ACP achieved better performance. Especially on ACP240, which contains a small amount of data, the CL-ACP model showed a better performance than the machine learning models. Although the results of AntiCP2.0 were better than those of other machine learning methods, its performance is based on feature construction, including location preference, which is a complex construction process and has certain limitations. The comparison results show that CL-ACP can extract high-quality features better than the machine learning models using the same features and coding methods. In addition, CL-ACP does not require manual feature design, and it has better robustness even when the amount of data is small.

**Table 4:** Performance of comparison models and CL-ACP on the ACP datasets

Dataset	Methods	Acc (%)	Sens (%)	Spec (%)	Prec (%)	Mcc (%)	AUC
ACP736	SVM	80.97	81.86	80.06	81.06	61.97	0.810
	RF	81.52	81.06	82.00	82.44	63.08	0.815
	NB	75.41	**90.13**	60.14	70.34	52.87	0.751
	PTPD	80.97	81.86	80.06	81.06	61.97	0.884
	ACP-DL	80.81	81.39	80.22	81.00	61.67	0.890
	AntiCP2.0	81.21	87.59	74.85	79.13	62.87	0.843
	iACP-DRLF	80.72	86.68	74.24	78.74	61.38	0.859
	CL-ACP	**83.83**	82.93	**84.76**	**85.15**	**67.86**	**0.909**
ACP240	SVM	79.58	83.01	75.61	80.22	59.59	0.793
	RF	81.66	84.58	78.30	82.05	63.48	0.814
	NB	70.83	88.40	50.43	67.35	43.01	0.694
	PTPD	79.58	83.01	75.61	80.22	59.59	0.784
	ACP-DL	83.75	88.40	78.45	83.16	68.29	0.903
	AntiCP2.0	84.00	88.64	76.16	84.18	71.19	0.894
	iACP-DRLF	84.11	88.01	74.35	84.03	70.35	0.903
	CL-ACP	**87.92**	**90.74**	**84.76**	**88.41**	**76.56**	**0.935**
ACP539	SVM	76.80	38.71	**97.70**	**89.88**	48.34	0.682
	RF	76.80	45.46	93.96	79.93	46.88	0.698
	NB	75.41	55.13	90.14	78.34	50.87	0.606
	PTPD	74.94	37.09	95.70	82.65	42.82	0.740
	ACP-DL	72.72	60.08	80.34	65.43	41.37	0.831
	AntiCP2.0	82.38	69.25	95.00	85.27	60.09	0.881
	iACP-DRLF	82.56	65.21	92.00	82.16	60.99	0.882
	CL-ACP	**84.41**	**77.48**	88.23	78.46	**65.98**	**0.921**

Bold indicates the highest value

Among the neural network models, ACP-DL, PTPD and iACP-DRLF were selected for comparison. We used fivefold cross-validation and the same evaluation indicators to evaluate the models. Considering that we used the datasets collected by Yi et al. as the benchmark datasets, we used ACP-DL as the main comparison method. On the ACP240 dataset, all indicators of CL-ACP were higher than those of the comparison models. Except for the sensitivity indicator, all indicators showed more than 4% improvement compared with ACP-DL, and the ROC value was significantly improved. On the ACP736 dataset, the sensitivity and ROC values were improved by approximately 2% compared with ACP-DL, and the other indicators were improved by more than 3%. The experimental results of PTPD and ACP-DL were worse than those of CL-ACP, mainly because CL-ACP's LSTM component can capture important sequence information. In contrast to the ACP-DL model, the CNN component of CL-ACP can capture ACP spatial information. Therefore, CL-ACP can be combined with neural networks with different structures to obtain sufficient characteristic information, which can be well applied to the identification and prediction of ACPs. The iACP-DRLF method performed well on both benchmark datasets. This mainly because it used two sequence embedding techniques and deep learning to characterize embedded sequences. However, sequence embedding required a high time cost, and the verification time of iACP-DRLF was the highest among all comparison methods (please see Additional file 5: Table S4).

To further verify the model prediction performance, we conducted a comparative experiment on ACP539 dataset, and the verification results are shown in Table 4. The average Acc of CL-ACP on the ACP539 dataset was 84.41%, the average Sens was 77.48%, the average Mcc was 65.98%, and the ROC value was 0.921, all of which were the highest among all comparison models. For the evaluation of unbalanced data, the Mcc value can be used to measure the classifier's quality, and the ROC value can measure overall model performance. It can be seen from Table 4 and Additional file 1: Figure S1 that the Mcc and ROC values of the machine learning models were lower among the comparison methods, and the ROC values of the neural network models were higher than those of the machine learning models. Moreover, the Mcc and ROC values of CL-ACP were the highest, indicating that CL-ACP still had a better performance when the data were slightly unbalanced.

It is worth noting that the specificity of each model was higher than its respective sensitivity for the ACP539 dataset because sensitivity and specificity are antagonistic to a certain extent [58]. This phenomenon also shows that these models missed some true positive samples. However, CL-ACP maintained high specificity with the highest sensitivity and better recognized true positive and true negative data. There are two main reasons. The first reason is that the ACP539 was unbalanced. The number of negative samples was about twice that of positive samples, leading to more false negatives predicted by the model. Another reason was that since the negative samples in the ACP539 dataset were composed of AMPs, which shared high similarity with ACPs [60]. This data-trained model may result in large false negatives for prediction. Especially when the dataset is small, the model will overfit the data and generate more false negative data, resulting in low sensitivity. As shown in Table 4, machine learning models have low sensitivity and high specificity because sensitivity and specificity are antagonistic. CL-ACP adopted a variety of regularization methods to avoid overfitting ACP539, especially negative samples, so fewer false negatives were generated compared with the machine learning models and obtained higher sensitivity. Moreover, sensitivity and specificity are antagonistic, so the true positive and false positive data of CL-ACP were both higher, resulting in lower specificity and accuracy.

### The performances of CL-ACP on antimicrobial peptide datasets

To verify the generalization ability of CL-ACP on short peptide data, we used other AMP datasets collected from APD and previous related works, including AAP, ABP, ACP, AIP, AVP, CPP, QSP, and PBP. Since most ACP data were contained in the ACP736 and ACP240 datasets, this procedure did not include testing on ACP datasets.

**Fig. 4:**
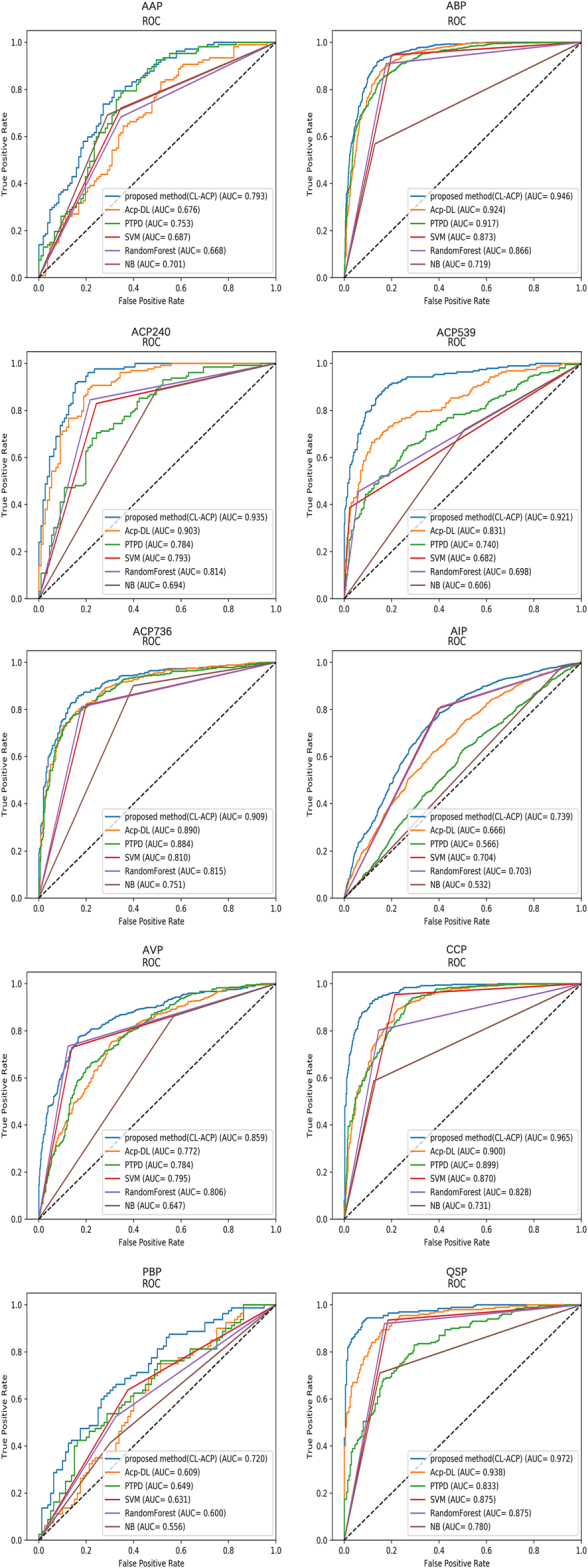
The ROC curves of the antimicrobial peptide datasets for CL-ACP and the comparison models

Figure 4 shows the ROC curves for various models. The AUC values of CL-ACP on ABP, CPP, and QSP were 0.945, 0.965, and 0.973, respectively, which were comparable to those on the ACP benchmark datasets, and these results were achieved by using only peptide sequence-level and structural information, as well as the advanced features automatically extracted by the effective architecture of CL-ACP.

The areas under the ROC curve of the AMP datasets AAP, ABP, ACP736, ACP240, ACP539, AIP, AVP, CPP, PBP and QSP for CL-ACP were 0.793, 0.946, 0.909, 0.935, 0.921, 0.739, 0.859, 0.965, 0.720 and 0.972, respectively. Compared with other models, the AUC values of CL-ACP were the highest, which further confirms the model generalizability and potential for the use of CL-ACP in AMP predictions. We will explore the incorporation of additional feature information to improve the model general AMP prediction performance in future work.

## Discussion

Experiments showed that CL-ACP had a good predictive performance and robustness relative to the comparison methods. In experiments on other AMPs, CL-ACP also showed better generalizability.

The performance of CL-ACP benefits from several major factors. (1) The peptide secondary structures contain key information about antitumour activity of ACPs, and the introduction of peptide secondary structures improves the feature richness. (2) The introduction of the parallel combined network model can fully extract local features and long-term dependence information from the feature space, effectively reduce the model complexity and prevent the problem of overfitting. (3) The introduction of the multi-head self-attention mechanism strengthens the representation of sequence information, as also indicated by its visualization. This is also the first attempt to introduce attention mechanism into ACP prediction.

## Conclusions

In this paper, we proposed an ACP prediction model constructed with a hybrid CNN and LSTM, termed CL-ACP. It used a multi-head self-attention mechanism to enhance the peptide sequence expression and incorporated peptide secondary structure characteristics to better characterize the feature space. CNN networks were used to obtain the local hidden characteristics of ACPs. The sequence dependence information of amino acid residues was captured by LSTM, which reduced information loss. Finally, the extracted advanced features were input to the fully connected layer for prediction. Comparative experiments on benchmark datasets showed that CL-ACP had a better predictive performance than existing prediction models, improving ACP identification. Comparative experiments on the ACP539 dataset showed that even when the negative data contained noise, the performance of CL-ACP was better than those of comparison models, indicating good robustness of CL-ACP. Comparative experiments on AMPs data showed that CL-ACP is not limited to the prediction of ACPs but can also automatically extract practical features. CL-ACP can learn efficient abstract representations of short peptide data to discover novel ACPs and AMPs, providing helpful information for drug development to treat various cancers and other diseases.

Although CL-ACP had a good performance in predicting ACPs, it still lacked meaningful biological explanation. For example, the multi-head self-attention mechanism can enhance weights of the essential residues in peptide sequences. However, the biological rationale is unclear and will therefore be a focus of future work. Simultaneously, we will consider effective feature fusion methods and model structures, such as capsule networks [58], to improve the performance of the model.

## Supplementary Information

**Additional file 1**.** Figure S1**.** a** ROC curves of ACP240 dataset on CL-ACP and comparison methods.** b** ROC curves of ACP736 dataset on CL-ACP and comparison methods.** c** ROC curves of ACP539 dataset on CL-ACP and comparison methods.

**Additional file 2**.** Table S1**. The results of CL-ACP using multiple numbers of head on the benchmark datasets.

**Additional file 3**.** Table S2**. Comparison results of using regularized and non-regularized multi-head self-attention mechanism.

**Additional file 4**.** Table S3**. k-fold cross-validation results of the proposed model CL-ACP on the benchmark datasets.

**Additional file 5**.** Table S4**. The amount of parameters and time spent on the 5-fold cross-validation of the model on the ACP datasets (The 5-fold cross-validation time of SVM, NB and RF and the validation time of AntiCP2.0 are very fast, so they are ignored).

**Additional file 6**.** Table S5**. The performance of two models of iACP_DRLF and AntiCP2.0 on ACP datasets.

## Data Availability

The datasets supporting the conclusions of this article are ACP datasets available from the previous studies and the Antimicrobial Peptide Database(APD) [12, 13, 22, 38]. The Amp datasets are from the studies of Balachandran et al. [39–45]. All data generated or analysed during this study can be obtained from https://github.com/zjlyn1314/CL-ACP/tree/main/Datasets.

## Abbreviations

AAC: Amino acid composition
Acc: Accuracy
AAP: Antiangiogenic peptide
ABP: Antibacterial peptide
ACP: Anticancer peptide
AIP: Anti-inflammatory peptide
Arg: Arginine
Asn: Asparagine
AVP: Antiviral peptide
AMP: Antimicrobial peptide
AUC: The area under ROC curve
ACP-DL: A deep learning long short-term memory model to predict anticancer peptides using high-efficiency feature representation
AntiCP 2.0: An updated model for predicting anticancer peptides
BILSTM: Bi-directional long short-term memory network
CPP: Cell penetrating peptide
CNN: Convolution neural network
CL-ACP: A parallel combination of CNN and LSTM Anticancer Peptide Recognition Model
Cys: Cysteine
DeepACLSTM: Deep asymmetric convolutional long short-term memory neural models for protein secondary structure prediction
FN: False negative
FP: False positive
Gly: Glycine
Glu: Glutamicacid
Gln: Glutarnine
His: Histidine
Ile: Isoleucine
Leu: Leucine
iACP-DRLF: Anticancer peptides prediction with deep representation learning features
LSTM: Long short-term memory network
Met: Methionine
MCC: Matthew’s correlation coefficient
NB: Naive bayesian model
Phe: Phenylalanine
PBP: Polystyrene surface binding peptide
Prec: Precision
PSI-BLAST: Position specific iterated BLAST
PSSM: Position specific scoring matrices
PTPD: Prediction of therapeutic peptide by deep learning and word2Vec
QSP: Quorum sensing peptide
RAAAC: Reduced amino acid alphabet
RAAC: Reduced amino acid composition
ReLU: Rectified linear unit
RF: Random forest
ROC: Receiver operating characteristic
Ser: Serine
SPIDER3: Capturing non-local interactions by long short term memory bidirectional recurrent neural networks for improving prediction of protein secondary structure
Sens: Sensitivity
Spec: Specificity
SVM: Support vector machine
Text-CNN: Text-convolutional neural network
TN: True negative
TP: True positive
Tyr: Tyrosine
Trp: Tryptophan

## References

[1] Domingues MM, Felício M, Gonalves S: Antimicrobial peptides: effect on bacterial cells: methods and protocols. Atomic Force Microsc.; 2019. 10.1007/978-1-4939-8894-5_13 30374871

[2] Shibue T, Weinberg RA: EMT, CSCs, and drug resistance: the mechanistic link and clinical implications. Nat Rev Clin Oncol. 2017. 10.1038/nrclinonc.2017.44 PMC5720366 28397828

[3] Barras D, Widmann C: Promises of apoptosis-inducing peptides in cancer therapeutics. Curr Pharm Biotechnol. 2011; 12(8). 10.2174/138920111796117337 21470140

[4] Pérez-Peinado C, Dias SA, Domingues MM, Benfield AH, Freire JM, Rádis-Baptista G, Gaspar D, Castanho M, Craik DJ, Henriques ST (2018). Mechanisms of bacterial membrane permeabilization by crotalicidin (Ctn) and its fragment Ctn(15–34), antimicrobial peptides from rattlesnake venom. J Biol Chem.

[5] Zafar S, Beg S, Panda SK, Rahman M, Ahmad FJ: Novel therapeutic interventions in cancer treatment using protein and peptide-based targeted smart systems. Semin Cancer Biol. 2019. 10.1016/j.semcancer.2019.08.023 31442570

[6] Sah BNP, Vasiljevic T, McKechnie S, Donkor ON: Identification of anticancer peptides from bovine milk proteins and their potential roles in management of cancer: a critical review. Compr Rev Food Sci Food Saf 2015; 14(2). 10.1111/1541-4337.12126 33401807

[7] Araste F, Abnous K, Hashemi M, Taghdisi SM, Ramezani M, Alibolandi M. Peptide-based targeted therapeutics: focus on cancer treatment. J Controlled Release 2018; 292:141–62. 10.1016/j.jconrel.2018.11.004 30408554

[8] Teerasak E-K, Pennapa T, Sittiruk R, Ladda M, Pramote C. Prediction of anticancer peptides against MCF-7 breast cancer cells from the peptidomes of Achatina fulica mucus fractions. Comput Struct Biotechnol J. 2016, 14. 10.1016/j.csbj.2015.11.005 PMC4706611 26862373

[9] Findlay F, Proudfoot L, Stevens C, Barlow PG (2016). Cationic host defense peptides; novel antimicrobial therapeutics against Category A pathogens and emerging infections. Pathog Global Health.

[10] Melicherčík P, Nešuta O, Čeřovský V. Antimicrobial peptides for topical treatment of osteomyelitis and implant-related infections: study in the spongy bone. Pharmaceuticals 2018. 10.3390/ph11010020 PMC5874716 29462909

[11] Hajisharifi Z, Piryai Ee M, Beigi MM, Behbahani M, Mohabatkar H (2014). Predicting anticancer peptides with Chou's pseudo amino acid composition and investigating their mutagenicity via Ames test. J Theor Biol.

[12] Tyagi A, Kapoor P, Kumar R, Chaudhary K, Gautam A, Raghava GPS: In silico models for designing and discovering novel anticancer peptides. Sci Rep. 2013, 3(1). 10.1038/srep02984 PMC6505669 24136089

[13] Wei C, Hui D, Pengmian F, Hao L, Kuo-Chen C. iACP: a sequence-based tool for identifying anticancer peptides. *Oncotarget* 2016, 7(13). 10.18632/oncotarget.7815 PMC4941358 26942877

[14] Haney EF, Mansour SC, Hancock R. Antimicrobial peptides: an introduction. Antimicrobial Peptides; 2017. 10.1007/978-1-4939-6737-7_1 28013493

[15] Huang Y, Qi F, Yan Q, Hao X, Chen Y: Alpha-helical cationic anticancer peptides: a promising candidate for novel anticancer drugs. Mini Rev Med Chem. 2015, 15(1). 10.2174/1389557514666141107120954 25382016

[16] Zandsalimi F, Talaei S, Ahari MN, Aghamiri S, Zadeh ZZ (2020). Antimicrobial peptides: a promising strategy for lung cancer drug discovery?. Expert Opin Drug Discov.

[17] Nhung D, Günther W, Lisa G, Mario S, Beate K, Christian KH, Monika S-K. Cationic membrane-active peptides - anticancer and antifungal activity as well as penetration into human skin. Exp Dermatol. 2014, 23(5). 10.1111/exd.12384 24661024

[18] Rhys H, Yang Y, Kuldip P, Zhou Y (2017). Capturing non-local interactions by long short term memory bidirectional recurrent neural networks for improving prediction of protein secondary structure, backbone angles, contact numbers, and solvent accessibility. Bioinformatics.

[19] Chen Y, Guarnieri MT, Vasil AI, Vasil ML, Mant CT, Hodges RS. Role of peptide hydrophobicity in the mechanism of action of alpha-helical antimicrobial peptides. Antimicrobial Agents Chemother. 2007; 51(4). 10.1128/AAC.00925-06 PMC1855469 17158938

[20] Chen Y, Vasil AI, Rehaume L, Mant CT, Burns JL, Vasil ML, Hancock R, Hodges RS. Comparison of biophysical and biologic properties of -helical enantiomeric antimicrobial peptides. Chem Biol Drug Des. 2006. 10.1111/j.1747-0285.2006.00349.x PMC3252236 16492164

[21] Hammami R, Fliss I. Current trends in antimicrobial agent research: chemo- and bioinformatics approaches. Drug Discovery Today 2010; 15(13). 10.1016/j.drudis.2010.05.002 20546918

[22] Leyi W, Chen Z, Huangrong C, Jiangning S, Ran S. ACPred-FL: a sequence-based predictor using effective feature representation to improve the prediction of anti-cancer peptides. Bioinformatics (Oxford, England) 2018; 34(23). 10.1093/bioinformatics/bty451 PMC6247924 29868903

[23] Akbar S, Hayat M, Iqbal M, Jan MA. iACP-GAEnsC: evolutionary genetic algorithm based ensemble classification of anticancer peptides by utilizing hybrid feature space. Artif Intell Med. 2017, 79. 10.1016/j.artmed.2017.06.008 28655440

[24] Agrawal P, Bhagat D, Mahalwal M, Sharma N, Raghava GP. AntiCP 2.0: an updated model for predicting anticancer peptides. Brief Bioinform. 2021, 22(3). 10.1093/bib/bbaa153 32770192

[25] Lv Z, Cui F, Zou Q, Zhang L, Xu L. Anticancer peptides prediction with deep representation learning features. Brief Bioinform. 2021. 10.1093/bib/bbab008 33529337

[26] Fenglin L, Minghui W, Yu L, Xing-Ming Z, Ao L: DeepPhos: prediction of protein phosphorylation sites with deep learning. Bioinformatics (Oxford, England) 2019, 35(16). 10.1093/bioinformatics/bty1051 PMC6691328 30601936

[27] Huiqing W, Yue M, Chunlin D, Chun L, Jingjing W, Dan L: CL-PMI: A precursor MicroRNA identification method based on convolutional and long short-term memory networks. Front Genet. 2019, 10. 10.3389/fgene.2019.00967 PMC6798641 31681416

[28] Guo Y, Li W, Wang B, Liu H, Zhou D. DeepACLSTM: deep asymmetric convolutional long short-term memory neural models for protein secondary structure prediction. BioMed Central 2019, 20(1). 10.1186/s12859-019-2940-0 PMC6580607 31208331

[29] Fei H, Rui W, Jiagen L, Lingling B, Dong X, Xiaowei Z. Large-scale prediction of protein ubiquitination sites using a multimodal deep architecture. BMC Syst Biol. 2018, 12(Suppl 6). 10.1186/s12918-018-0628-0 PMC6249717 30463553

[30] Zheng X, Fu X, Wang K, Wang M. Deep neural networks for human microRNA precursor detection. BMC Bioinform. 2020, 21(1). 10.1186/s12859-020-3339-7 PMC6958766 31931701

[31] Yi H-C, You Z-H, Zhou X, Cheng L, Li X, Jiang T-H, Chen Z-H. ACP-DL: a deep learning long short-term memory model to predict anticancer peptides using high efficiency feature representation. Mol Ther - Nucleic Acids 2019. 10.1016/j.omtn.2019.04.025 PMC6554234 31173946

[32] Zhang D, Xu H, Su Z, Xu Y. Chinese comments sentiment classification based on word2vec and SVM perf. Expert Syst Appl 2015; 42(4).

[33] Chuanyan W, Rui G, Yusen Z, Yang DM. PTPD: predicting therapeutic peptides by deep learning and word2vec. BMC Bioinform 2019; 20(1). 10.1186/s12859-019-3006-z PMC6728961 31492094

[34] Kim Y. Convolutional neural networks for sentence classification. *Eprint Arxiv* 2014.

[35] Jeff D, Anne HL, Marcus R, Subhashini V, Sergio G, Kate S, Trevor D. Long-term recurrent convolutional networks for visual recognition and description. IEEE Trans Pattern Anal Mach Intell 2017; 39(4). 10.1109/TPAMI.2016.2599174 27608449

[36] Lin Z, Feng M, Santos C, Yu M, Xiang B, Zhou B, Bengio Y. A structured self-attentive sentence embedding. 2017.

[37] Li W, Godzik A. Cd-hit: a fast program for clustering and comparing large sets of protein or nucleotide sequences. Bioinformatics 2006, 22(13). 10.1093/bioinformatics/btl158 16731699

[38] Boopathi V, Subramaniyam S, Malik A, Lee G, Yang DC. mACPpred: a support vector machine-based meta-predictor for identification of anticancer peptides. Int J Mol Sci 2019. 10.3390/ijms20081964 PMC6514805 31013619

[39] Tramontano A, Ramaprasad AE, Singh S, Gajendra P, Venkatesan S. AntiAngioPred: a server for prediction of anti-angiogenic peptides. PLoS ONE 2015; 10(9):e0136990 10.1371/journal.pone.0136990 PMC4559406 26335203

[40] Sharma BK, Sneh L, Gps R (2007). Analysis and prediction of antibacterial peptides. BMC Bioinform..

[41] Balachandran M, Shin TH, Kim MO, Gwang L. AIPpred: sequence-based prediction of anti-inflammatory peptides using random forest. Front Pharmacol. 2018, 9:276. 10.3389/fphar.2018.00276 PMC5881105 29636690

[42] Nishant T, Abid Q, Manoj K (2012). AVPpred: collection and prediction of highly effective antiviral peptides. Nucleic Acids Res.

[43] Wei L, Xing P, Ran S, Shi G, Quan Z: CPPred-RF: a sequence-based predictor for identifying cell-penetrating peptides and their uptake efficiency. J Proteome Res. 2017, 16(5). 10.1021/acs.jproteome.7b00019 28436664

[44] Akanksha R, Kumar GA, Manoj K, Lukasz K. Prediction and analysis of quorum sensing peptides based on sequence features. Plos One 2015, 10(3):e0120066. 10.1371/journal.pone.0120066 PMC4363368 25781990

[45] Li N, Kang J, Jiang L, He B, Hao L, Huang J. PSBinder: a web service for predicting polystyrene surface-binding peptides. BioMed Res Int. 2017; (2017-12-27), 2017:1–5. 10.1155/2017/5761517 PMC5763211 29445741

[46] Prashant K, Jayachandran K, Suzana S. Antimicrobial peptides: diversity, mechanism of action and strategies to improve the activity and biocompatibility in vivo. Biomolecules 2018; 8(1):4. 10.3390/biom8010004 PMC5871973 29351202

[47] Lombardi L, Stellato MI, Oliva R, Falanga A, Galdiero M, Petraccone L, D’Errico G, Santis AD, Galdiero S, Vecchio PD. Antimicrobial peptides at work: interaction of myxinidin and its mutant WMR with lipid bilayers mimicking the *P. aeruginosa* and *E. coli* membranes. Sci Rep. 2017; 7:44425. 10.1038/srep44425 PMC5353584 28294185

[48] Amos S, Vermeer LS, Ferguson PM, Kozlowska J, Davy M, Bui TT, Drake AF, Lorenz CD, Mason AJ (2016). Antimicrobial peptide potency is facilitated by greater conformational flexibility when binding to gram-negative bacterial inner membranes. Sci Rep.

[49] Berthony D, Di YP (2017). Antimicrobial peptides with selective antitumor mechanisms: prospect for anticancer applications. Oncotarget.

[50] Wimley WC. How does Melittin Permeabilize membranes? Biophys J. 2018, 114(2). 10.1016/j.bpj.2017.11.3738 PMC5984972 29401422

[51] Sani MA, Separovic F: How membrane-active peptides get into lipid membranes. Acc Chem Res. 2016:1130–1138. 10.1021/acs.accounts.6b00074 27187572

[52] Lehmann J, Retz M, Sidhu SS, Suttmann H, Sell M, Paulsen F, Harder J, Unteregger G, Stöckle M. Antitumor activity of the antimicrobial peptide Magainin II against Bladder Cancer Cell Lines. Eur Urol. 2006, 50(1) 10.1016/j.eururo.2005.12.043 16476519

[53] He H, Garcia EA (2009). Learning from imbalanced data. IEEE Trans Knowl Data Eng.

[54] Vaswani A, Shazeer N, Parmar N, Uszkoreit J, Jones L, Gomez AN, Kaiser L, Polosukhin I. Attention is all you need. *arXiv* 2017.

[55] Voita E, Talbot D, Moiseev F, Sennrich R, Titov I. Analyzing multi-head self-attention: specialized heads do the heavy lifting, the rest can be pruned. In Meeting of the Association for Computational Linguistics: 2019.

[56] Jian L, Tu Z, Tong Z. Multi-head attention with disagreement regularization. In *Proceedings of the 2018 Conference on Empirical Methods in Natural Language Processing: 2018*.

[57] Srivastava N, Hinton G, Krizhevsky A, Sutskever I, Salakhutdinov R (2014). Dropout: a simple way to prevent neural networks from overfitting. J Mach Learn Res.

[58] Duolin W, Yanchun L, Dong X. Capsule network for protein post-translational modification site prediction. Bioinformatics 2019; 35(14). 10.1093/bioinformatics/bty977 PMC6612812 30520972

[59] Ning Q, Zhao X, Bao L, Ma Z, Zhao X (2018). Detecting Succinylation sites from protein sequences using ensemble support vector machine. BMC Bioinform..

[60] Vijayakumar S, Ptv L (2015). ACPP: a web server for prediction and design of anti-cancer peptides. Int J Pept Res Ther.

