# Supplementary figures and images for: CL-ACP: a parallel combination of CNN and LSTM anticancer peptide recognition model
Source: BMC Bioinformatics. 2021 Oct 20;22:512. doi: 10.1186/s12859-021-04433-9 (PMC8527680; doi:10.1186/s12859-021-04433-9)

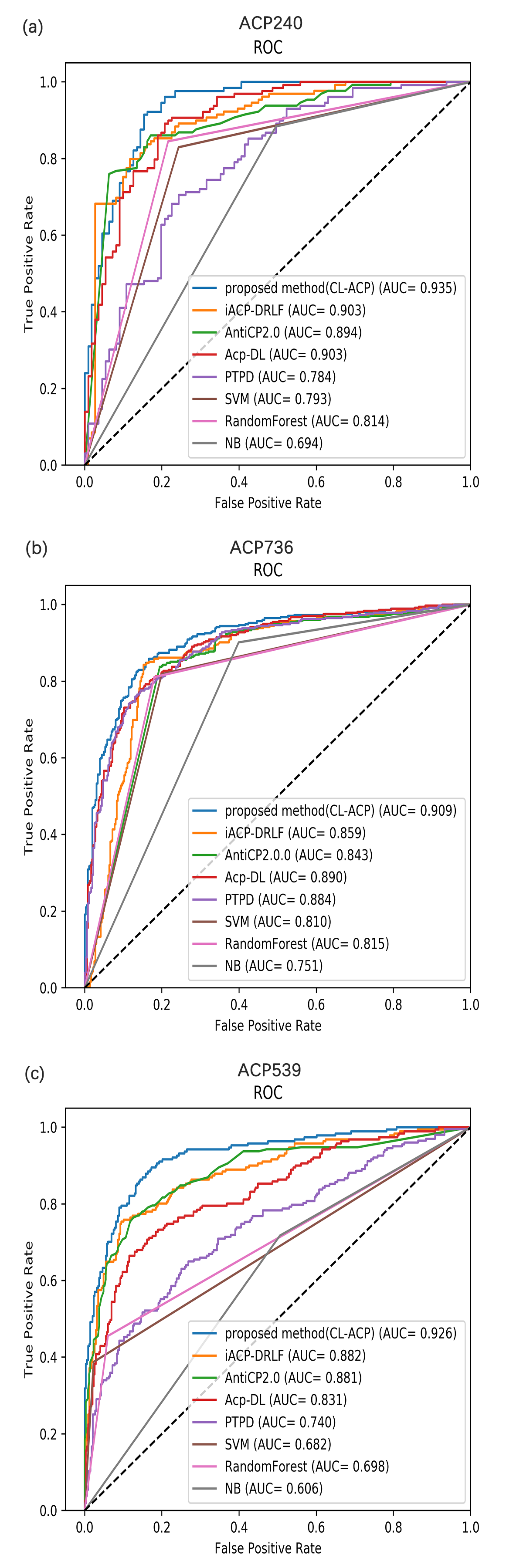

Supplement: Supplementary file 1 — Additional file 1. Figure S1. a ROC curves of ACP240 dataset on CL-ACP and comparison methods. b ROC curves of ACP736 dataset on CL-ACP and comparison methods. c ROC curves of ACP539 dataset on CL-ACP and comparison methods. [file 12859_2021_4433_MOESM1_ESM.tiff]
