# Supplementary material for: CL-ACP: a parallel combination of CNN and LSTM anticancer peptide recognition model
Source: BMC Bioinformatics. 2021 Oct 20;22:512. doi: 10.1186/s12859-021-04433-9 (PMC8527680; doi:10.1186/s12859-021-04433-9)
Supplement: Supplementary file 2 — Additional file 2. Table S1. The results of CL-ACP using multiple numbers of head on the benchmark datasets. [file 12859_2021_4433_MOESM2_ESM.docx]

**Table S1.** The results of CL-ACP using multiple numbers of head on the benchmark datasets.

| Dataset | Heads | Acc(%) | Sens(%) | Spec(%) | Pre(%) | MCC | AUC | Time(m) |
| --- | --- | --- | --- | --- | --- | --- | --- | --- |
| ACP736 | 1 | 81.69 | 82.27 | 81.11 | 81.95 | 65.46 | 0.899 | 1.5 |
|  | **2** | **83.83** | **82.93** | **84.77** | **85.15** | **67.86** | **0.909** | 2.0 |
|  | 4 | 83.83 | 82.00 | 83.66 | 84.40 | 67.82 | 0.906 | 2.5 |
|  | 8 | 83.15 | 82.67 | 83.67 | 84.30 | 66.56 | 0.905 | 4.0 |
|  | 16 | 83.66 | 82.86 | 82.00 | 83.38 | 67.08 | 0.904 | 7.1 |
| ACP240 | 1 | 85.83 | 89.96 | 81.23 | 85.84 | 72.56 | 0.914 | 1.07 |
|  | **2** | **87.92** | **90.74** | 84.74 | **88.41** | **76.56** | **0.935** | 1.4 |
|  | 4 | 87.33 | 89.96 | **85.56** | 86.51 | 75.12 | 0.932 | 1.6 |
|  | 8 | 86.08 | 89.20 | 84.74 | 88.09 | 74.72 | 0.930 | 2.6 |
|  | 16 | 86.67 | 88.46 | 84.74 | 87.88 | 73.88 | 0.929 | 3.9 |
