# Supplementary material for: CL-ACP: a parallel combination of CNN and LSTM anticancer peptide recognition model
Source: BMC Bioinformatics. 2021 Oct 20;22:512. doi: 10.1186/s12859-021-04433-9 (PMC8527680; doi:10.1186/s12859-021-04433-9)
Supplement: Supplementary file 3 — Additional file 3. Table S2. Comparison results of using regularized and non-regularized multi-head self-attention mechanism. [file 12859_2021_4433_MOESM3_ESM.docx]

**Table S2.** Comparison results of using regularized and non-regularized multi-head self-attention mechanism

| Dataset | Method | Heads | Acc(%) | Sens(%) | Spec(%) | Pre(%) | MCC | AUC |
| --- | --- | --- | --- | --- | --- | --- | --- | --- |
| ACP240 | ordinary | **2** | **87.92** | **90.74** | 84.74 | **88.41** | **76.56** | **0.935** |
|  |  | 4 | 87.33 | 89.96 | **85.56** | 86.51 | 75.12 | 0.932 |
|  |  | 8 | 86.08 | 89.20 | 84.74 | 88.09 | 74.72 | 0.930 |
|  |  | 16 | 86.67 | 88.46 | 84.74 | 87.88 | 73.88 | 0.929 |
|  | regularized | 2 | **87.82** | 89.93 | 85.88 | 87.95 | 76.12 | **0.934** |
|  |  | 4 | 87.08 | **90.40** | 84.57 | 85.01 | 75.26 | 0.931 |
|  |  | 8 | 87.42 | 89.63 | 85.89 | **89.00** | **76.18** | 0.932 |
|  |  | 16 | 87.95 | 89.97 | **86.88** | 87.51 | 76.05 | **0.934** |
| ACP736 | ordinary | 2 | **83.83** | **82.93** | **84.77** | **85.15** | **67.86** | **0.909** |
|  |  | 4 | 83.83 | 82.00 | 83.66 | 84.40 | 67.82 | 0.906 |
|  |  | 8 | 83.15 | 82.67 | 83.67 | 84.30 | 66.56 | 0.905 |
|  |  | 16 | 83.66 | 82.86 | 82.00 | 83.38 | 67.08 | 0.904 |
|  | regularized | 2 | 83.58 | 83.01 | 83.27 | 85.05 | 67.35 | 0.907 |
|  |  | 4 | **84.03** | 82.25 | **84.96** | **85.40** | 67.60 | **0.909** |
|  |  | 8 | 84.05 | 82.17 | 84.67 | 84.30 | 67.56 | 0.908 |
|  |  | 16 | 83.96 | **83.86** | 83.00 | 85.28 | **67.88** | **0.909** |
