# Supplementary material for: CL-ACP: a parallel combination of CNN and LSTM anticancer peptide recognition model
Source: BMC Bioinformatics. 2021 Oct 20;22:512. doi: 10.1186/s12859-021-04433-9 (PMC8527680; doi:10.1186/s12859-021-04433-9)
Supplement: Supplementary file 4 — Additional file 4. Table S3. k-fold cross-validation results of the proposed model CL-ACP on the benchmark datasets. [file 12859_2021_4433_MOESM4_ESM.docx]

**Table S3.** k-fold cross-validation results of the proposed model CL-ACP on the benchmark datasets

| Dataset | k-fold cross-validation | Acc(%) | Sens(%) | Spec(%) | Prec(%) | Mcc(%) | AUC |
| --- | --- | --- | --- | --- | --- | --- | --- |
|  | CL-ACP(k=5) | 83.83 | 82.93 | 84.76 | 85.15 | 67.86 | 0.909 |
| ACP736 | CL-ACP (k=6) | 83.30 | 84.56 | 81.99 | 83.09 | 66.76 | 0.906 |
|  | CL-ACP (k=8) | 83.83 | 84.28 | 83.42 | 84.35 | 67.91 | 0.905 |
|  | CL-ACP (k=10) | 85.32 | 85.58 | 85.05 | 85.82 | 70.80 | 0.910 |
|  | CL-ACP (k=5) | 87.92 | 90.74 | 84.72 | 88.41 | 76.56 | 0.935 |
| ACP240 | CL-ACP (k=6) | 85.83 | 88.38 | 82.89 | 85.84 | 71.56 | 0.930 |
|  | CL-ACP (k=8) | 87.50 | 89.15 | 85.65 | 87.98 | 75.07 | 0.933 |
|  | CL-ACP (k=10) | 87.50 | 90.77 | 83.86 | 87.38 | 75.59 | 0.928 |
|  | CL-ACP (k=5) | 84.41 | 77.48 | 88.23 | 78.46 | 65.98 | 0.923 |
| ACP539 | CL-ACP (k=6) | 85.34 | 76.41 | 90.21 | 81.31 | 67.70 | 0.920 |
|  | CL-ACP (k=8) | 84.59 | 74.32 | 90.26 | 81.24 | 66.17 | 0.921 |
|  | CL-ACP (k=10) | 86.26 | 78.49 | 90.53 | 82.17 | 69.89 | 0.922 |
