# Supplementary material for: CL-ACP: a parallel combination of CNN and LSTM anticancer peptide recognition model
Source: BMC Bioinformatics. 2021 Oct 20;22:512. doi: 10.1186/s12859-021-04433-9 (PMC8527680; doi:10.1186/s12859-021-04433-9)
Supplement: Supplementary file 5 — Additional file 5. Table S4. The amount of parameters and time spent on the 5-fold cross-validation of the model on the ACP datasets (The 5-fold cross-validation time of SVM, NB and RF and the validation time of AntiCP2.0 are very fast, so they are ignored). [file 12859_2021_4433_MOESM5_ESM.docx]

**Table S4.** The amount of parameters and time spent on the 5-fold cross-validation of the model on the ACP datasets(The 5-fold cross-validation time of SVM, NB and RF and the validation time of AntiCP2.0 are very fast, so they are ignored)

| Dataset | Methods | Running time(minutes) | Total params |
| --- | --- | --- | --- |
| ACP240 | CL-ACP(sk) | 1.42 | 63986 |
|  | CL-ACP(ca) | 6.18 | 138217 |
|  | ACP-DL | 0.48 | 313743 |
|  | PTPD | 3.33 | 239851 |
|  | iACP_DRLF(only verification) | 12.73 | None |
| ACP736 | CL-ACP(sk) | 2.01 | 63986 |
|  | CL-ACP(ca) | 7.89 | 138217 |
|  | ACP-DL | 0.78 | 313743 |
|  | PTPD | 10.08 | 239851 |
|  | iACP_DRLF(only verification) | 27.58 | None |
| ACP539 | CL-ACP(sk) | 1.78 | 63986 |
|  | CL-ACP(ca) | 6.59 | 138217 |
|  | ACP-DL | 0.62 | 313743 |
|  | PTPD | 7.77 | 239851 |
|  | iACP_DRLF(only verification) | 20.12 | None |
| **CL-ACP(sk) means that the multi-head self-attention mechanism with skip-connection; CL-ACP(ca) indicates that the multi-head self-attention mechanism adopts cascade mode.** | | | |
