# Supplementary material for: CL-ACP: a parallel combination of CNN and LSTM anticancer peptide recognition model
Source: BMC Bioinformatics. 2021 Oct 20;22:512. doi: 10.1186/s12859-021-04433-9 (PMC8527680; doi:10.1186/s12859-021-04433-9)
Supplement: Supplementary file 6 — Additional file 6. Table S5. The performance of two models of iACP_DRLF and AntiCP2.0 on ACP datasets. [file 12859_2021_4433_MOESM6_ESM.docx]

**Table S5** The performance of two models of iACP_DRLF and AntiCP2.0 on ACP datasets

| Dataset | Model | Acc(%) | Prec(%) | Sens(%) | Spec(%) | Mcc(%) | AUC | |
| --- | --- | --- | --- | --- | --- | --- | --- | --- |
| ACP736 | AntiCP2.0(a) | 81.21 | 79.13 | 87.59 | 74.85 | 62.87 | 0.843 | |
|  | iACP-DRLF(a) | 80.72 | 78.74 | 86.68 | 74.24 | 61.38 | 0.859 | |
|  | AntiCP2.0(b) | 76.27 | 76.66 | 85.08 | 61.15 | 48.15 | 0.759 | |
|  | iACP-DRLF(b) | 74.30 | 73.78 | 85.55 | 61.61 | 47.38 | 0.715 | |
| ACP240 | AntiCP2.0(a) | 84.00 | 84.18 | 88.64 | 76.16 | 71.19 | 0.894 | |
|  | iACP-DRLF(a) | 84.11 | 84.03 | 88.01 | 74.35 | 70.35 | 0.903 | |
|  | AntiCP2.0(b) | 81.58 | 82.35 | 86.55 | 75.80 | 63.37 | 0.872 | |
|  | iACP-DRLF(b) | 74.71 | 71.67 | 86.84 | 49.69 | 61.64 | 0.802 | |
| ACP539 | AntiCP2.0(b) | 82.38 | 85.27 | 69.25 | 95.00 | 60.09 | 0.881 | |
|  | iACP-DRLF(b) | 82.56 | 82.16 | 65.21 | 92.00 | 60.99 | 0.882 | |
|  | AntiCP2.0(a) | 71.36 | 69.80 | 45.66 | 66.37 | 43.18 | 0.694 | |
|  | iACP-DRLF(a) | 69.74 | 74.41 | 41.99 | 70.47 | 46.03 | 0.702 | |
| **a** represents the pre-trained model of the dataset composed of ACPs (positive) and non-ACPs (negative); **b** represents the pre-trained model of the dataset composed of ACPs (positive) and AMPs (negative) with unknown anti-tumour activity. | | | | | | | |  |
